# 

**Published:** 1878-01

**Authors:** 


### Contents of January Number.

### ORIGINAL.

ART. I.—Epithelioma of the Cervix Uteri. Amputation by Prof. James P. White, M. D. Report of case by Edward N. Brush, M. D., ........ 201
ART. II.—Adenitis, Acute and Chronic. By J. R. Larzelere, M.D., . 204
ART. III.—Removal of the Thyroid Gland. By Prof. Julius F. Miner, M. D. Reported by Chas. A. Wall, B. S., ........ 207

### MISCELLANEOUS.

The Plaster of Paris Jacket in Spinal Diseases. By E. Andrews, A. M., M. D., ........ 210
Salicylic Acid in Rheumatism, ........ 214
Medical Certificates in the Daily Papers, ........ 215
Elementary Advice to Mothers and Nurses, ........ 218
Poison of Putrefied Blood, ........ 219
The Prophylactic Treatment of Placenta Prævia, ........ 220
Arsenic in Wall-Papers and Dresses, ........ 221
Are Ants Civilized? ........ 222
International Congress of the Medical Sciences. Held at Geneva, Switzerland, Sept. 9-15, 1877 ........ 223
Composition of Secret Remedies, ........ 227
A Case in Proof of Non-Identity of Variola and Varicella, ........ 228
Shall Medical Witnesses Receive a Proper Compensation? ........ 229
Military Surgery in Turkey, ........ 230
United States Medical Library, ........ 230

### OBITUARY.

Dr. Edward H. Clarke, of Boston, Mass., ........ 230
George Ayer, M. D., ........ 231

### EDITORIAL.

The Struggle For Existence Becomes General, ........ 232
The Popular Science Monthly, ........ 236
Books Reviewed:
Ziemssen's Cyclopœdia. Vol. XVI., ........ 236
Lectures on Fevers. Loomis, ........ 237
Transactions of the New York Pathological Society. Vol. II. 238
Cutaneous and Venereal Memoranda. Piffard and Fox, ........ 238
Personal Appearance. Sozinsky, ........ 239
Outlines of Modern Chemistry, ........ 239
Books and Pamphlets Received, ........ 240

